# Supplementary material for: Effect of the Ketone Body, D-β-Hydroxybutyrate, on Sirtuin2-Mediated Regulation of Mitochondrial Quality Control and the Autophagy–Lysosomal Pathway
Source: Cells. 2023 Feb 2;12(3):486. doi: 10.3390/cells12030486 (PMC9914182; doi:10.3390/cells12030486)
Supplement: Supplementary file 1 [file cells-12-00486-s001.zip › cells-2142784-supplementary.pdf]

Supplementary Table S1. List of primers.

Gen	# of NCBI reference	Primers 5´-3´ (forward and reverse)
Lamp1	NM_012857.2	CCACGTTCAGCACCTCCA<br>GACCCAAACCTGTCACTTTCTAC
Lamp2	XM_006257486.4	AGCCCAATGCCTAACACATC<br>CTGCCCTCCCAATAACAAGA
Ctsb	NM_022597.2	AGGCTGGACGCAACTTCTAC<br>ACTGTTCCCGTGCATCAAA
Ctsd	NM_134334.3	CCTGGGCGATGTCTTTATTG<br>GCAAAGCCGACCCTATT
Atg5	NM_001014250.2	ATATGAAGGCACACCCCTGA<br>GTGAGCCTCAACTGCATCCT
Rab7	NM_023950.4	GGCCTTCTACAGAGGTGCAG<br>TCTTTGTGGCCACTTGTCTG
Map1lc3a	NM_199500.2	GACCGGCCTTTCAAGCAG<br>CCTTGTAGCGCTCGATGAT
Sqstm1	NM_175843.5	CGCTTCAGCTTCTGCTTCAG<br>GGCCATTGTCAGTTCCTCAT
Pgc1α	NM_031347.1	GTGCAGCCAAGACTCTGTATGG<br>GTCCAGGTCATTCACATCAAGTTC
Tfam	NM_031326.2	GAAAGCACAAATCAAGAGGAG<br>CTGCTTTTCATCATGAGACAG
Tuba1a	NM_022298.1	GATCTGATGTATGCCAAGCG<br>TCCACAGAATCCACACCAAC

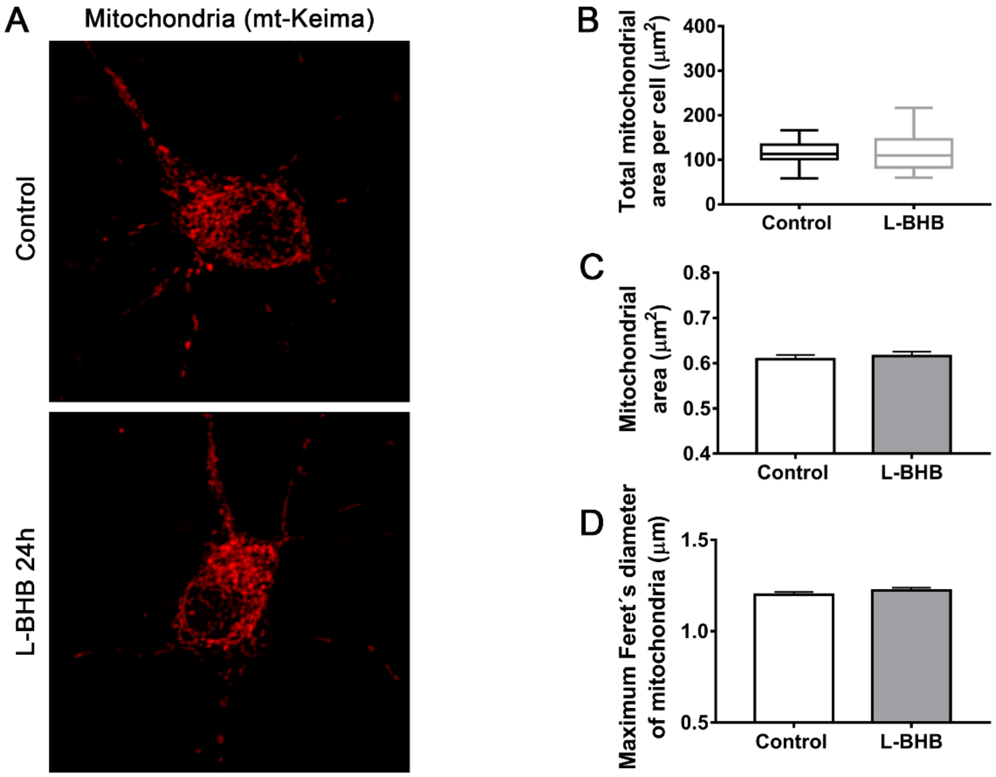

Supplementary Figure S1. Effect of L-BHB on mitochondrial morphology. **A**) Representative micrographs of neurons transfected with mkeima-red-mito (mt-keima) and treated and no-treated with 5 mM L-BHB for 24 h. **B**) Quantification of total mitochondrial area per cell, **C**) individual mitochondrial area and **D**) maximum Feret´s diameter of mitochondria. Box-plot data (B) are expressed as median (line), 25th and 75th percentiles (bound of box), minimum and maximum values (whiskers) from 23 cells of 3 independent experiments. Data of C and D are expressed as Mean ± SEM from 3 independent experiments and were analyzed by one-way ANOVA followed by Fisher's post-hoc test. Data of B were analyzed by Kruskal-Wallis test followed by Dunn´s post hoc test.

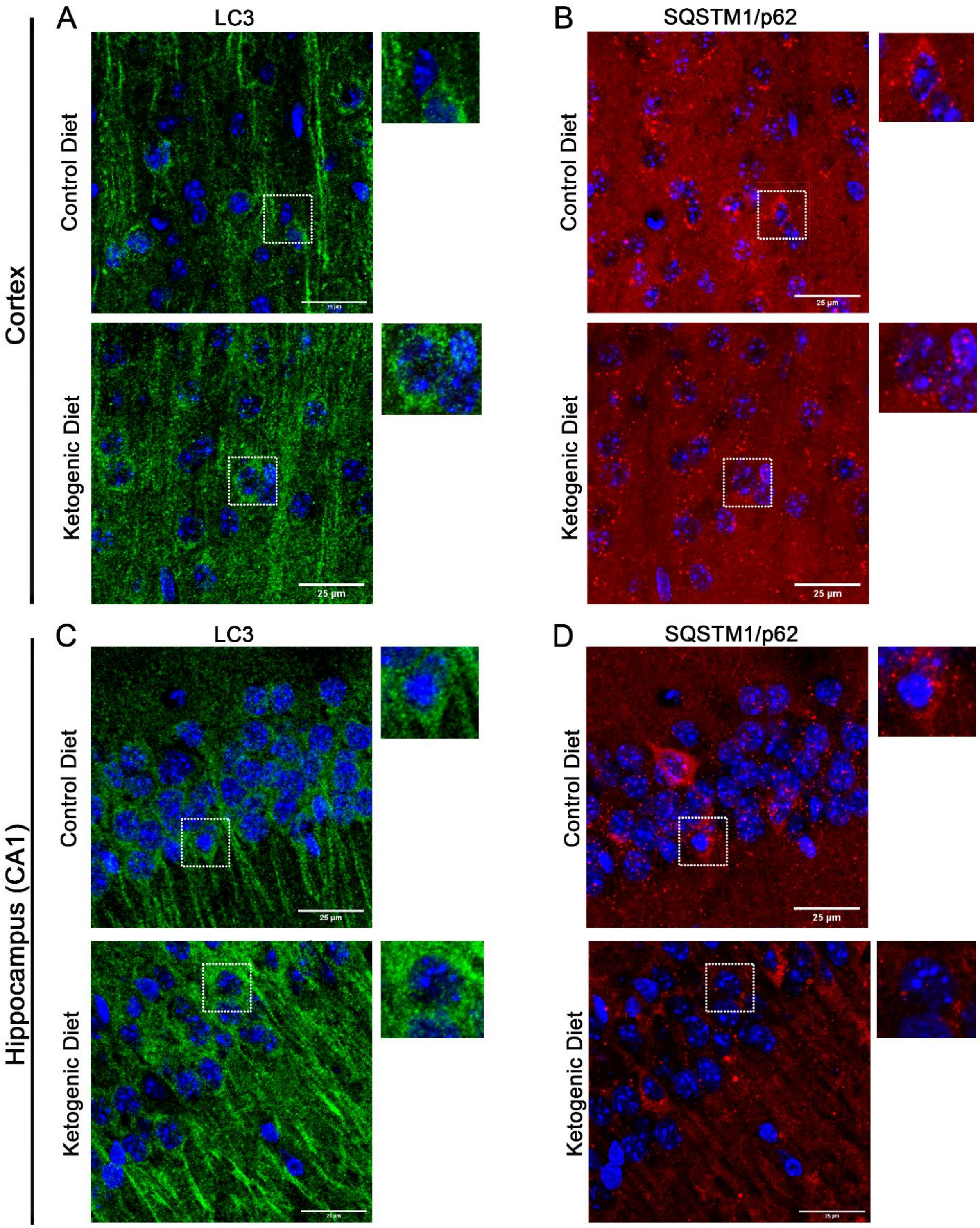

Supplementary Figure S2. Changes in autophagy markers in the brain of mice treated with control diet or ketogenic diet. Representative immunofluorescence of LC3 (**A**, **C**) and SQSTM1/p62 (**B**, **D**) in the cortex (A, B) and the hippocampus (C, D) of mice treated for 2 weeks with control diet (CD) or ketogenic diet (KD).

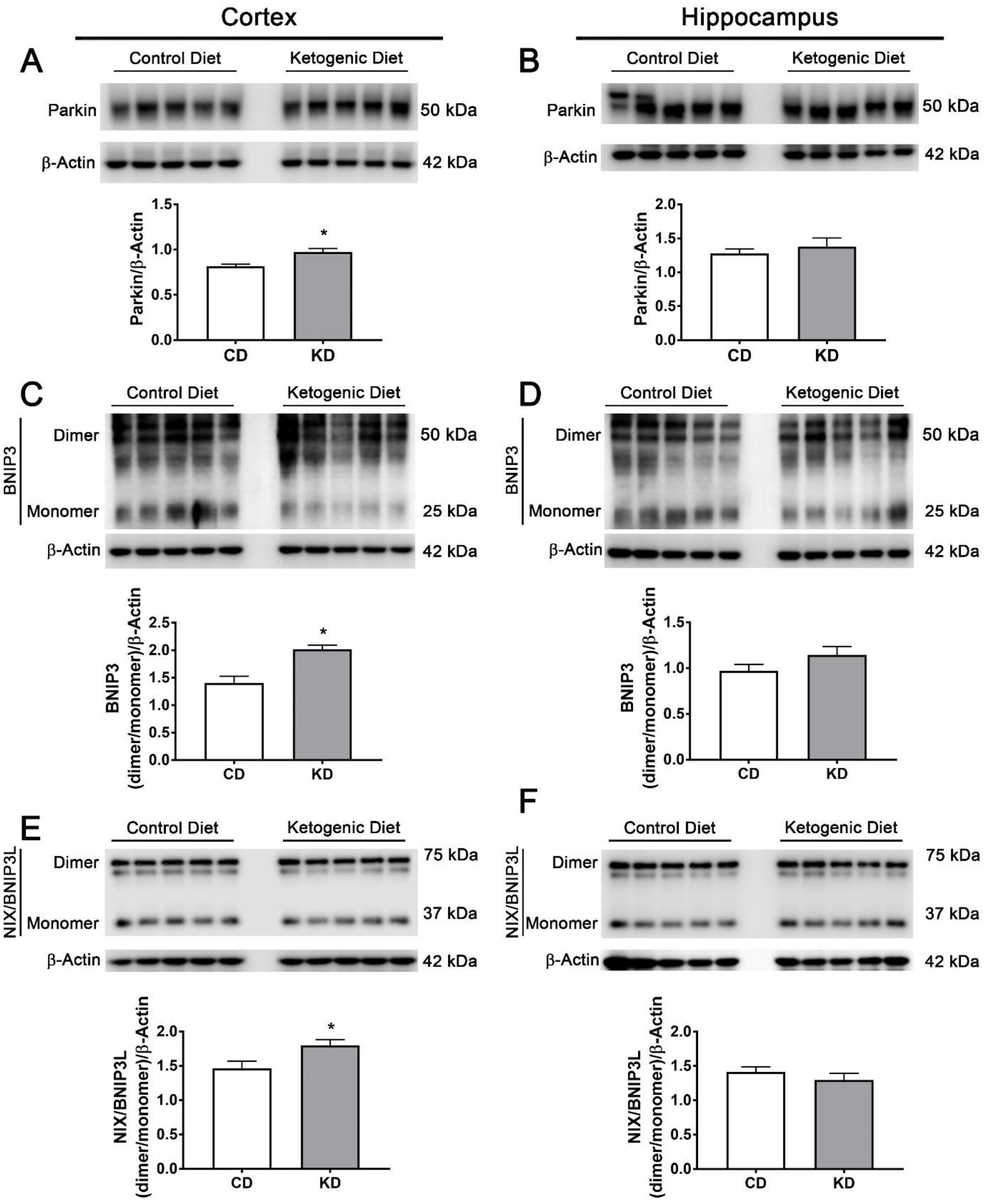

Supplementary Figure S3. Changes in mitophagy markers in the brain of mice treated with control diet or ketogenic diet. Representative Western blot and quantification of A and B) Parkin/*β*-actin, C and D) BNIP3 (dimer/monomer)/*β*-actin and E and F) NIX/BNIP3L (dimer/monomer)/*β*-actin in the cortex (A, C, E) and the hippocampus (B, D, F) of mice treated for 2 weeks with control diet (CD) or ketogenic diet (KD). Data are expressed as Mean ± SEM from 5 individuals per group. Data were analyzed by Student´s *t*-test. *$p<0.05$ vs control diet.
